# Visual pollution real images benchmark dataset on the public roads

**DOI:** 10.1016/j.dib.2023.109491

**Published:** 2023-08-10

**Authors:** Mohammad AlElaiwi, Mugahed A. Al-antari, Hafiz Farooq Ahmad, Areeba Azhar, Badar Almarri, Jamil Hussain

**Affiliations:** a Computer Science Department, College of Computer Sciences and Information Technology (CCSIT), King Faisal University, P.O. Box 400, Al-Ahsa, 31982, Saudi Arabia; b Department of Artificial Intelligence, College of Software & Convergence Technology, Daeyang AI Center, Sejong University, Seoul, 05006, Korea; c Department of Mathematics, College of Natural & Agricultural Sciences, University of Califor-nia-Riverside (UCR), Riverside, CA, USA; d Department of Data Science, College of Software & Convergence Technology, Daeyang AI Center, Sejong University, Seoul, 05006, Korea

**Keywords:** Artificial intelligence, Computer vision, Pollution, Machine learning, Active learning, Image classification, Deep learning

## Abstract

The term quality of life (QoL) refers to a wide range of multifaceted concepts that often involve subjective assessments of both positive and negative aspects of life. It is difficult to quantify QoL as the word has varied meanings in different academic areas and may have different connotations in different circumstances. The five sectors most commonly associated with QoL, however, are Health, Education, Environmental Quality, Personal Security, Civic Engagement, and Work-Life Balance. An emerging issue that falls under environmental quality is visual pollution (VP) which, as detailed in this study, refers to disruptive presences that limit visual ability in public roads with an emphasis on excavation barriers, potholes, and dilapidated sidewalks. Quantifying VP has always been difficult due to its subjective nature and lack of a consistent set of rules for systematic assessment of visual pollution. This emphasizes the need for research and module development that will allow government agencies to automatically predict and detect VP. Our dataset was collected from different regions in the Kingdom of Saudi Arabia (KSA) via the Ministry of Municipal and Rural Affairs and Housing (MOMRAH) as a part of a VP campaign to improve Saudi Arabia's urban landscape. It consists of 34,460 RGB images separated into three distinct classes: excavation barriers, potholes, and dilapidated sidewalks. To annotate all images for detection (i.e., bounding box) and classification (i.e., classification label) tasks, the deep active learning strategy (DAL) is used where an initial 1,200 VP images (i.e., 400 images per class) are manually annotated by four experts. Images with more than one object increase the number of training object ROIs which are recorded to be 8,417 for excavation barriers, 25,975 for potholes, and 7,412 for dilapidated sidewalks. The MOMRAH dataset is publicly published to enrich the research domain with the new VP image dataset.

Specifications Table

**Table:** 

Subject	• Artificial Intelligence • Computer Vision and Pattern Recognition • Applied Machine Learning
Specific subject area	• The evident degradation and poor aesthetic quality of natural and man-made environments is referred to as visual pollution (VP). • It also refers to any disruptive event that restricts people's mobility on public routes, such as excavation obstacles, potholes, and deteriorating sidewalks. • The VP dataset is gathered from the Kingdom of Saudi Arabia (KSA). • All images in the dataset are labeled, to perform AI supervised detection and classification techniques and construct the suggested deep learning framework.
Type of data	• RGB Images: The dataset contains 34,460 VP RGB images categorized into three classes: ① Excavation barriers (6,322 images), ② Potholes (21,429 images), ③ Dilapidated sidewalks (6,709 images). • Labels or Annotations for: ① Detection Labels: bounding box coordinators of each object inside the image: start point (x_1_,y_1_), endpoint (x_2_,y_2_), width (w), and height (h). ② Classification Labels: all objects in a single image are annotated by four experts by providing the associated class label for each image.
How the data were acquired	• As part of an effort to reduce visual pollution and enhance Saudi Arabia's urban environment, the dataset was gathered from the kingdom of Saudi Arabia (KSA) by the Ministry of Municipal and Rural Affairs and Housing (MOMRAH). • Saudi nationals and foreigners are requested to use their cellphones to capture images of visual pollution and upload them to the official “Balady” Mobile Application in order to compile this dataset. • We received formal government approval to use and disseminate this dataset for our research [Permission letters Nos. 4300454067 & 4400003688]. This collection contains 34,460 photos divided into three categories: excavation barriers, potholes, and dilapidated sidewalks.
Data format	• Type: Raw RGB images with three channels: Red (R), Green (G), and blue (B). • Format: Joint Photographic Experts Group “jpeg or jpg”.
Description of data collection	• Collection: The raw data images were supplied by users of the Balady Mobile Application as part of the Ministry of Municipal and Rural Affairs and Housing's (MOMRAH) visual pollution initiative to enhance Saudi Arabia's urban landscape. • Preprocessing: The data obtained is occasionally erroneous, inconsistent, and may not reflect specific behaviors or patterns. Images must thus be preprocessed into a format that the machine learning algorithm can use to create the model. The vast majority of pictures are classified as visual pollution, but there are also outlier images that are irrelevant. Manual intervention was required to detect these ``outliers'' and exclude them from the model's training and testing. • Data Splitting: The dataset is randomly divided into 70% for training, 10% for validation, and 20% for testing and validating the suggested AI models for visual pollution prediction. • Data Labelling: To further enhance our framework, all objects inside a single image must be annotated for both detection and classification tasks. For detection tasks, the labels must be represented as a bounding box composed of the start point (x_1_,y_1_), end point (x_2_,y_2_), width (w), and height (h). • Deep Active Learning (DAL) Strategy: Because labeling is difficult and time intensive, we employed the deep active learning (DAL) technique to annotate the remaining images. The following are the steps in the deep active learning strategy. First, we chose the clearest 400 images from each class and asked four experts to manually annotated the object localization using the CVAT toolkit [1]. Second, based on the annotated sample dataset, the optimal deep learning model is chosen to train the 400 images per class. Finally, the trained DL model is utilized to test the remaining photos. This process was placed in a loop to review and certify the automatic labeling process.
Data source location	• Institution: Ministry of Municipal and Rural Affairs and Housing (MOMRAH). • City/Town/Region: All Kingdom regions • Country: Saudi Arabia
Data accessibility	• Repository name: Saudi Arabia Public Roads Visual Pollution Dataset. • Data identification number (DOI): 10.17632/bb7b8vtwry.2 • Direct URL to data: https://data.mendeley.com/datasets/bb7b8vtwry
Related research article	• Published Article: MDPI and ACS StyleAlElaiwi, M.; Al-antari, M.A.; Ahmad, H.F.; Azhar, A.; Almarri, B.; Hussain, J. VPP: Visual Pollution Prediction Framework Based on a Deep Active Learning Approach Using Public Road Images. Mathematics 2023, 11, 186. https://doi.org/10.3390/math11010186AMA StyleAlElaiwi M, Al-antari MA, Ahmad HF, Azhar A, Almarri B, Hussain J. VPP: Visual Pollution Prediction Framework Based on a Deep Active Learning Approach Using Public Road Images. Mathematics. 2023; 11(1):186. https://doi.org/10.3390/math11010186Chicago/Turabian StyleAlElaiwi, Mohammad, Mugahed A. Al-antari, Hafiz Farooq Ahmad, Areeba Azhar, Badar Almarri, and Jamil Hussain. 2023. ``VPP: Visual Pollution Prediction Framework Based on a Deep Active Learning Approach Using Public Road Images'' Mathematics 11, no.1:186. https://doi.org/10.3390/math11010186

## 1 Value of the Data

• Introducing efficient business processes to optimize the visual pollution inspection process.
• Reducing visual pollution operations load through better usage of the Data Clustering Engine (DCE).
• Designing, re-designing or updating processes that can benefit from AI engines’ outputs to maximize efficiency.
• Incorporating new KPIs to increase visibility on inspection performance.
• Highly useful for machine learning experts working in the field of automatic classification and detection of visual pollution across a number of different landscapes.
• Used for various purposes like risk-based dispatching which can guide inspectors to areas with the highest potential for visual pollution. Case prioritization can also provide visibility on case priority based on criticality and visibility factors while case clustering can identify the duplicated reporting of individual visual pollution cases.

## 2 Objective

The scarcity of annotated visual pollution images in a multi-class manner for both detection and classification tasks are always a challenge for supervised AI models. A new private VP dataset is collected by the Ministry of Municipal and Rural Affairs and Housing (MOMRAH), Saudi Arabia. This dataset has various VP classes and is called the MOMRAH benchmark dataset: excavation barriers, potholes, and dilapidated sidewalks. With the data made accessible in this article, we aim to contribute to filling the existing knowledge gap and provide an annotated ready dataset for further studies and research. As well as assisting government organizations and decision makers with dataset to process on their AI-based technology that automatically predicts and recognizes visual pollution without user intervention. An end-to-end visual pollution prediction (VPP) framework based on the deep active learning (DAL) approach was proposed to simultaneously detect and classify visual pollutants using the published dataset. The proposed framework was architected around the following steps: real VP dataset collection, pre-processing, DAL approach for automatic data annotation, data splitting and augmentation, and simultaneous VP detection and classification [2].

## 3 Data Description

**Fig. 1:**
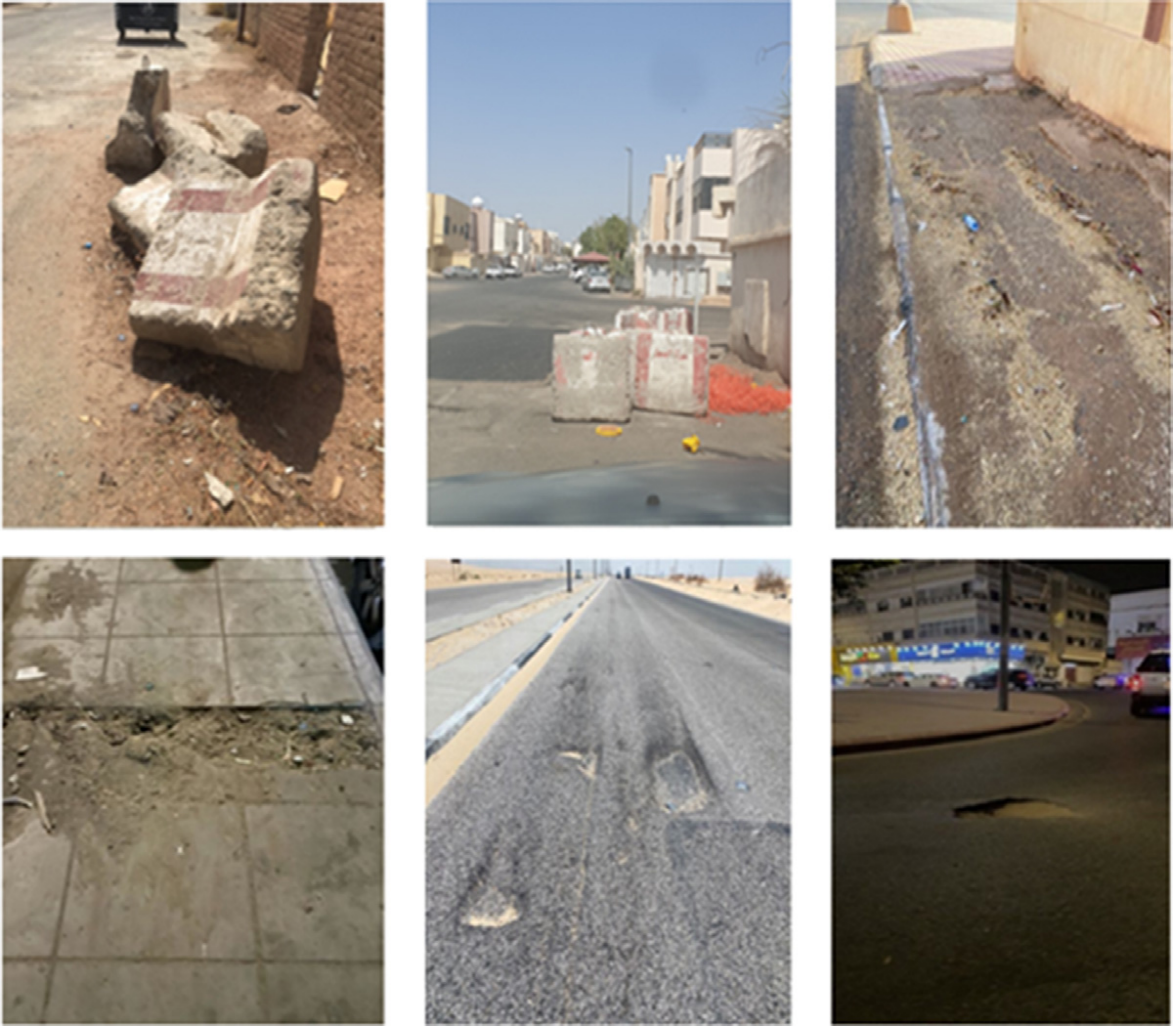
Examples of the published dataset.

The real VP dataset is collected from the kingdom of Saudi Arabia (KSA) regions via the Ministry of Municipal and Rural Affairs and Housing (MOMRAH). This dataset is collected form Urban public roads in KSA during the research period starting May 2021 till January 2022. Visual pollution appears in digital images with varying irregular shapes, colors, and sizes, as observed in Fig. 1. This dataset is called “Saudi Arabia Public Roads Visual Pollution Dataset” and it contains 34,460 VP images which are divided over three different classes: 6,322 for excavation barriers, 21,429 for potholes, and 6,709 for dilapidated sidewalks. The dataset per class is split into 70% for training, 10% for validation, and 20% for testing.

To prepare the dataset the following pre-processing steps are performed: irrelevant image removal, normalization, resizing, and data splitting. The raw RGB images in the VP dataset are first carefully analyzed by experts wherein all irrelevant, inaccurate, or unreliable images to the visual pollution topic are immediately excluded. As the normalization process may improve the overall prediction performance, we normalized all images to bring their intensity in the range of [0, 255] [10, 11].

The uniqueness of proposed VP dataset is summarized as follows,

• Collecting a road visual pollution dataset is unique because it involves capturing and analyzing specific visual elements that contribute to pollution on roads.
• This dataset can provide valuable insights into the types and extent of visual pollution, such as billboards, signage, graffiti, or litter, affecting road environments.
• By collecting and analyzing this data, it can help policymakers, urban planners, and researchers understand the impact of visual pollution on road safety, aesthetics, and quality of life.
• This unique dataset can facilitate targeted interventions, design improvements, and policy decisions to mitigate visual pollution and create more visually appealing and sustainable road environments.

**Fig. 2:**
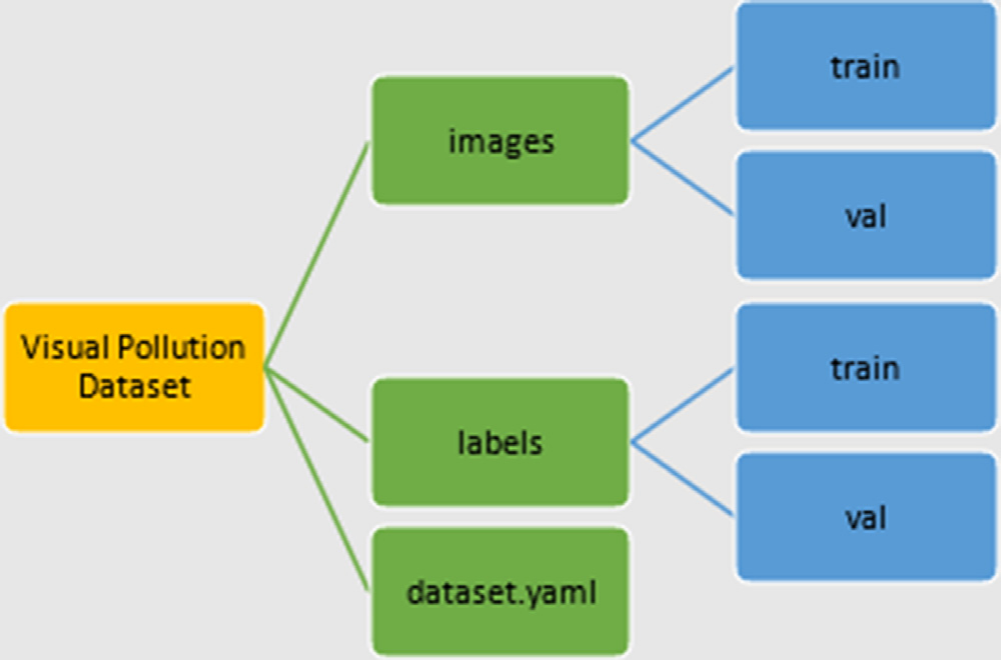
Directory structure of the dataset.

Fig. 2 show the directory structure of the dataset uploaded to Mendeley site. This is to be easier for researchers in the field to use and investigate it for further study.

## 4 Experimental Design, Materials and Methods

There are several ways of building an object detection model. Implementing an existing model architecture, for instance, is a swift and efficient method that may also provide guidance from prior implementations. This technique also benefits from transfer learning, a method that uses weights from previously built models and re-purposes them for new tasks. Alternatively, a model can also be built from scratch. Although this approach is time-consuming and requires further resources for training, it allows the programmer to maintain greater control over the model itself. The most difficult and time-consuming phases in this approach are data collecting and data pre-processing; availability, bias, quality, and legality are common challenges faced during these two crucial steps. Feature extraction approaches, training and testing of the ML algorithm, and model performance evaluations are also covered in this study.

The initial step in the machine learning pipeline and process flow is to acquire sufficient data for training the machine learning model. Our data was obtained by the Ministry of Municipal and Rural Affairs and Housing (MOMRAH) from various regions across the Kingdom of Saudi Arabia (KSA) as part of a visual pollution effort to enhance Saudi Arabia's urban landscape. To gather this dataset, Saudi nationals and expats were asked to capture potential visual pollutants and upload them to the government-created Balady mobile application [3]. The MOMRAH in Saudi Arabia granted our team formal authorization to utilize the data collected on Balady for this study. The VP real dataset is named ``Saudi Arabia Public Roads Visual Pollution Dataset'', and it contains 34,460 RGB photos for three separate classes: excavation barriers, potholes, and dilapidated sidewalks. The total number of training object ROIs recorded per class is 8,417 for excavation barriers, 25,975 for potholes, and 7,412 for dilapidated sidewalks. Moreover, to counteract the lack of labels on this raw dataset, all images were annotated for detection (i.e., bounding box) and classification (i.e., classification label) tasks using the deep active learning strategy where an initial 1,200 VP images (i.e., 400 images per class) were manually annotated by four experts. The dataset has been made available in order to supplement the research area with new VP-based datasets.

### 4.1 Data pre-processing

Raw data gathered by end users via the Balady Mobile Application are occasionally erroneous, unreliable, and not reflective of specific behaviors or patterns. As a result, photos must be preprocessed into a format acceptable for the machine learning algorithm to use and create a model. Although most images are considered to contain elements of visual pollution, there are also 'outlier images' that are irrelevant to our study. Manual intervention was required to detect these ``outlier pictures'' and exclude them from model training and testing.

The following pre-processing steps are performed to prepare the dataset for fine-tuning the deep learning models within the proposed framework: irrelevant image removal, normalization, resizing, and data splitting. Because the dataset is collected by individuals' mobile phone camera settings, the preprocessing normalization and resizing techniques are applied to be sure about the unique histogram distribution for all images. Meanwhile, the experts thoroughly examined the raw RGB images in the MOMRAH VP dataset, and irrelevant, erroneous, or untrustworthy images linked to the visual pollution problem are instantly deleted. As normalization can increase overall prediction accuracy, all images are adjusted to have intensities between [0, 255] [22,23]. Additionally, all images are also resized using bi-cubic interpolation to scale their intensity pixels into the range of 460 × 600.

### 4.2 Deep active learning (DAL) for automatic data annotation

Active learning is an efficient ML technique that identifies important data for manual annotation, therefore decreasing the number of man-hours required while also maximizing a model's performance. Data annotation itself, however, can be very costly in regard to the object detection operation. Each detection frame often has tens of thousands of pixels, which annotators must manually identify by placing boxes around corresponding objects. In addition to the high cost, monitoring and managing the quality of the annotations is also a challenge. To summarize, semi-supervision allows for improved general object detection systems, a multitude of challenges may arise when attempting to control the quality of annotations.

This technique starts by training a baseline model on a small, labeled dataset, which is subsequently applied to a corresponding unlabeled dataset. Using multiple query selection algorithms (random, uncertainty, and more), it evaluates whether each unlabeled sample has critical information that the baseline model has not yet learned. After the trained model has selected and labeled the samples containing the most significant information and they have been confirmed by a human, the samples may be added to the initial dataset to train a new model that is expected to perform better. Our proposed deep learning VPP framework detects and classifies VP objects into three categories: excavation barriers, potholes, and dilapidated sidewalks. To train and build this framework, every image in the dataset was annotated for detection and classification tasks. The detection label was expressed as a bounding box to encompass the entire object (i.e., ROI) within the image with coordinates at the start point (x_1_,y_1_), end point (x_2_,y_2_), width (w), and height (h). As this labeling procedure can be difficult and time-consuming, the deep active learning (DAL) approach was utilized to automatically annotate the remaining images in the VP dataset. This strategy was implemented as follows. First, we chose 400 of the clearest images from each class to be manually annotated for object localization using the CVAT toolset [1]. Second, based on the annotated sample dataset, the optimal deep learning detector model was chosen (i.e., 400 images per class). Third, among the remaining unlabeled photos, the trained model was then used to test for the most relevant images which are then finalized via the Query strategy and evaluated by an expert-in-loop. This selection technique is often carried out by examining strong similarities between the initial samples in the first round and the remaining unlabeled samples. The instances with the highest resemblance are placed in a loop to be systematically examined, modified, and confirmed by an expert. Once all images have been manually verified, the AI model is retrained using this new updated dataset of labelled images (400+ images in the confirmed subset). Images with lower similarity ratios that were unable to be labeled in the first round are utilized as a testing set in the DAL cycle's second round. The automated DAL procedure is therefore continued till the halting requirements are met by appropriately annotating all VP images. Following the completion of the DAL process and the creation of a benchmark dataset, the photos per class are randomly divided into three sets: 70% for training, 10% for validation, and 20% for testing. The training and validation sets are then used to train and fine-tune the AI models, while the evaluation strategy is performed using the isolated testing set.

## Ethics Statements

Not applicable.

## CRediT authorship contribution statement

**Mohammad AlElaiwi:** Conceptualization, Data curation, Investigation, Methodology, Software, Writing – review & editing. **Mugahed A. Al-antari:** Conceptualization, Data curation, Methodology, Software, Validation, Writing – original draft, Writing – review & editing. **Hafiz Farooq Ahmad:** Funding acquisition, Project administration, Supervision, Validation, Writing – review & editing. **Areeba Azhar:** Writing – review & editing. **Badar Almarri:** Validation, Writing – review & editing. **Jamil Hussain:** Conceptualization, Data curation, Investigation, Methodology, Software, Validation, Visualization, Writing – review & editing.

## Declaration of Competing Interest

The authors declare that they have no known competing financial interests or personal relationships that could have appeared to influence the work reported in this paper.

## Data Availability

Saudi Arabia Public Roads Visual Pollution Dataset (Original data) (Mendeley Data).
